# Antimicrobial activities of *Bacillus velezensis* strains isolated from stingless bee products against methicillin-resistant *Staphylococcus aureus*

**DOI:** 10.1371/journal.pone.0251514

**Published:** 2021-05-11

**Authors:** Mohamad Malik Al-adil Baharudin, Mohamad Syazwan Ngalimat, Fairolniza Mohd Shariff, Zetty Norhana Balia Yusof, Murni Karim, Syarul Nataqain Baharum, Suriana Sabri

**Affiliations:** 1 Enzyme and Microbial Technology Research Center, Faculty of Biotechnology and Biomolecular Sciences, Universiti Putra Malaysia, Serdang, Selangor, Malaysia; 2 Department of Microbiology, Faculty of Biotechnology and Biomolecular Sciences, Universiti Putra Malaysia, Serdang, Selangor, Malaysia; 3 Department of Biochemistry, Faculty of Biotechnology and Biomolecular Sciences, Universiti Putra Malaysia, Serdang, Selangor, Malaysia; 4 Department of Aquaculture, Faculty of Agriculture, Universiti Putra Malaysia, Serdang, Selangor, Malaysia; 5 Laboratory of Marine Biotechnology, Institute of Bioscience, Universiti Putra Malaysia, Serdang, Selangor, Malaysia; 6 Metabolomics Research Laboratory, Institute of Systems Biology, Universiti Kebangsaan Malaysia, Bangi, Malaysia; University of Messina, ITALY

## Abstract

Infections caused by methicillin-resistant *Staphylococcus aureus* (MRSA) have reached epidemic proportions globally. Therefore, there is an urgent need for a continuous supply of antibiotics to combat the problem. In this study, bacteria initially identified as species belonging to the *Bacillus amyloliquefaciens* operational group were re-identified based on the housekeeping gene, *gyrB*. Cell-free supernatants (CFS) from the strains were used for antimicrobial tests using the agar well diffusion assay against MRSA and various types of pathogenic bacteria. The minimum inhibitory concentration (MIC), minimum bactericidal concentration (MBC) and physicochemical characteristics of the CFS were determined. Based on *gyrB* sequence analysis, five strains (PD9, B7, PU1, BP1 and L9) were identified as *Bacillus velezensis*. The CFS of all *B*. *velezensis* strains showed broad inhibitory activities against Gram-negative and -positive as well as MRSA strains. Strain PD9 against MRSA ATCC 33742 was chosen for further analysis as it showed the biggest zone of inhibition (21.0 ± 0.4 mm). The MIC and MBC values obtained were 125 μl/ml. The crude antimicrobial extract showed bactericidal activity and was stable at various temperatures (40–80°C), pH (4–12), surfactants (Tween 20, Tween 80, SDS and Triton X-100) and metal ions (MgCI_2,_ NaCI_2,_ ZnNO_3_ and CuSO_4_) when tested. However, the crude extract was not stable when treated with proteinase K. All these properties resembled the characteristics of peptides. The antimicrobial compound from the selected strain was purified by using solvent extraction method and silica gel column chromatography. The purified compound was subjected to High Performance Liquid Chromatography which resulted in a single peak of the anti-MRSA compound being detected. The molecular weight of the anti-MRSA compound was determined by using SDS-PAGE and zymogram. The size of the purified antimicrobial peptide was approximately ~ 5 kDa. The antimicrobial peptide produced from *B*. *velezensis* strain PD9 is a promising alternative to combat the spread of MRSA infections in the future.

## Introduction

Diseases caused by bacterial infections associated with mortalities and morbidities have increased from time to time. The phenomenon is due to the emergence of antibiotic resistant bacteria that makes therapeutic options to treat the infections become more limited. Pathogens commonly associated with antibiotic resistance are termed as ‘ESKAPE’ pathogens which comprise of *Enterococcus faecium*, *Staphylococcus aureus*, *Klebsiella pneumoniae*, *Acinetobacter baumannii*, *Pseudomonas aeruginosa* and *Enterobacter* spp. [1]. Among all the pathogens, methicillin-resistant *Staphylococcus aureus* (MRSA) has been listed by the World Health Organization (WHO) in the high priority group based on the urgency for new antibiotics to treat them [1].

MRSA are opportunistic pathogens that are resistant to various antibiotics particularly penicillin class antibiotics [2–4]. MRSA are known as the common causes of nosocomial infections responsible for skin lesions, abscess and potentially fatal diseases such as pneumonia, osteomyelitis, severe sepsis and endocarditis fasciitis and toxinoses such as toxic shock syndrome [5]. Due to the dramatic increase in the resistance acquired by these bacteria, the death rate caused by MRSA infection is expected to surpass cancer in the coming years [6]. MRSA can also cause various infections in animal and poultry, such as lethal systemic infection in rabbit, bumble food in poultry and the most economically disastrous one; mastitis in dairy cattle and ruminants [2].

Natural sources are reservoirs for microorganisms that are capable of producing vast spectrums of antimicrobial metabolites [7]. Bacteria in the genus *Bacillus* are widely reported to produce broad-spectrums of antimicrobial metabolites [8]. The *B*. *amyloliquefaciens* operational group was reported to encode at least 4–5% of their genetic materials for antimicrobial metabolite production [9, 10]. Several anti-MRSA metabolites were identified from the *B*. *amyloliquefaciens* operational group, such as aminoglycoside antibacterial substance from *B*. *velezensis* RP147 [11], bacillusin A from *B*. *amyloliquefaciens* AP183 [12], phenolic compounds from *B*. *amyloliquefaciens* MHB1 [13], cyclic dipeptide, cyclo(L-leucyl-L-prolyl) from *B*. *amyloliquefaciens* MMS-50 [14], cyclic lipopeptides, such as surfactin, iturin and fengycin A from *B*. *amyloliquefaciens* JN68 [15], amysin from *B*. *amyloliquefaciens* SP-1-13LM [16] and antimicrobial peptide, CSpK14 from *B*. *amyloliquefaciens* K14 [17].

Recently, antibacterial activities were detected from species which belonged to the *B*. *amyloliquefaciens* operational group isolated from stingless bee, *Heterotrigona itama*, products [18]. However, there is still no information on the inhibition activity of the isolated strains against MRSA. Hence, further reassessment on the identification of the isolated strains is needed as the 16S rRNA gene failed to differentiate species within the *B*. *amyloliquefaciens* operational group which consisted of *B*. *amyloliquefaciens*, *B*. *velezensis* and *B*. *siamensis* [10, 19]. However, housekeeping genes such as *gyrB*, *rpoB* and *trpB* were useful in identifying the bacterial species under the *B*. *amyloliquefaciens* operational group [20]. Thus, this study aimed to identify and discover the potential of the strains from the *B*. *amyloliquefaciens* operational group as anti-MRSA. Here, the DNA gyrase subunit B gene, *gyrB* was used as another phylogenetic marker to further identify the bacterial species. Moreover, the antibacterial activities of crude extracts from the strains against MRSA were evaluated and their physicochemical characteristics were determined. Purification of the antimicrobial metabolite was done and its molecular weight ascertained. The data obtained could establish the antimicrobial metabolite from *B*. *velezensis* strains as a potential anti-MRSA metabolite.

## Materials and methods

### Bacterial strains, media and growth conditions

All the bacterial species (*Clostridium perfringens*, *Bacillus cereus*, *Serratia marcescens*, *Pseudomonas aeruginosa* and *Alcaligenes faecalis*) used in the screening of antimicrobial activities were obtained from the Department of Microbiology, Faculty of Biotechnology and Biomolecular Science, Universiti Putra Malaysia (UPM), Malaysia. *Vibrio parahaemolyticus*, *Vibrio alginolyticus* and *Aeromonas hydrophila* were obtained from the Department of Aquaculture, Faculty of Agriculture, UPM, Malaysia. All bacterial strains (PD9, B7, PU1, BP1 and L9) that belonged to the *B*. *amyloliquefaciens* operational group isolated from stingless bee, *Heterotrigona itama*, products [18] were obtained from the Enzyme and Microbial Technology Research Centre (EMTech, UPM, Malaysia). MRSA strains (ATCC 33742, ATCC 700699, ATCC 25922 and ATCC 33741) and MSSA strain ATCC 12228 were obtained from the Department of Biomedical Science, Faculty of Medicine and Health Science, UPM, Malaysia. Cultures were grown in 10 ml of nutrient broth (Merck, Darmstadt, Germany) at 37°C with shaking at 200 rpm for 24 h. For long-term storage, cultures were preserved in nutrient broth using 20% (v/v) glycerol at -80°C.

### *gyrB* sequence analysis

Bacterial genomic DNA was extracted from 16 h cultures by using the Wizard^®^ Genomic DNA Purification Kit (Promega, Madison, WI, USA) following the manufacturer’s instructions. The *gyrB* was amplified using polymerase chain reaction (PCR) with primers 22f (5’-GAAGTTATCATGACGGTACTTC-3’) and 1240r (5’-AGCGTACGAATGTGAGAACC-3’). The primers were used to amplify an approximately 1.2-kbp segment of *gyrB* [21]. Each 25 μl of PCR mixture contained 12.5 μl of 2 x PCR Taq Master Mix (Applied Biological Materials Inc, Richmond, BC, Canada), 0.5 μM of each primer and 100 ng of genomic DNA as a template. Thermal cycling was performed in a G-Storm GS1 thermal cycler (GRI Ltd., Essex, UK) with the following parameters: initial denaturation step of 94°C for 3 min, followed by 35 cycles of 94°C for 30 s, 60°C for 30 s and 72°C for 1 min. A final extension step consisting of 72°C for 5 min was included. The PCR products were checked by 1.5% (w/v) agarose gel electrophoresis and then were sent for sequencing (MyTACG Bioscience Enterprise, Kuala Lumpur, Malaysia). The sequences were analyzed with the Chromas Lite software (version 2.6.4; Technelysium Pty Ltd., South Brisbane, QLD, Australia) and compared against the sequences in the National Centre for Biotechnology Information (NCBI) nonredundant database by using the BLASTn program (https://www.ncbi.nlm.nih.gov/).

The phylogenetic analysis was done using the MEGA 7.0 software [22]. Type strains including *B*. *velezensis* CBMB205, *B*. *siamensis* KCTC 13613 and *B*. *amyloliquefaciens* DSM7 were included in the analysis. The nucleotide sequences were retrieved from the NCBI Entrez Genome Project database (https://www.ncbi.nlm.nih.gov/genome). Multiple sequence alignments were generated using the ClustalX programme [23]. The phylogenetic tree was obtained according to the neighbor-joining method [24] with the *p*-distance method [25] and 1,000 bootstraps [26]. *B*. *subtilis* subsp. *subtilis* 168 was used as the outgroup.

### Antibacterial activities of *B*. *velezensis* strains against various bacteria

The antibacterial activities of *B*. *velezensis* strains were examined against test bacteria by using the agar well diffusion assay with slight modifications [27]. Briefly, 100 μl of indicator bacterial strain suspension (bacterial suspension turbidity was compared to the 0.5 McFarland standard) were swabbed onto nutrient agar plate (Merck, Germany). About 6 mm diameter well was cut using the back of sterile 1-ml tip. The cell-free supernatant used for the test was obtained by centrifugation of 20 h bacterial cultures at 8 000 x *g* for 10 min. The well was filled with 100 μl CFS and incubated at 37°C. Chloramphenicol 30 μg/ml was used as the positive control. Zone of inhibition was observed and measured on the lawn culture plate after 24 h of incubation. The antibacterial activity screening was performed in triplicates.

### Minimum Inhibitory Concentration (MIC)

MIC was determined by using the microplate dilution method with slight modifications [28]. The CFS used in the experiment was obtained by growing strain PD9 alone as well as co-culture of strain PD9 and MRSA ATCC 33742 in nutrient broth (Merck, Germany) for 20 h. In the strain PD9 alone flask, 1 ml of strain PD9 culture at final cell density 10^3^ cfu/ml was inoculated into 99 ml nutrient broth. In the co-culture flask, 1 ml of strain PD9 and 1 ml of MRSA ATCC 33742 at final cell density 10^3^ cfu/ml were inoculated into 98 ml nutrient broth. The culture was centrifuged at 8 000 x *g* for 10 min to obtained the CFS. The CFS was serially diluted with nutrient broth by two-fold dilution (2^−1^ to 2^−10^). Then, 5 μl of MRSA ATCC 33742 cell suspension (standardized to 10^7^ cfu/ml) was deposited into each well. The 96-well plates were incubated aerobically at 37°C overnight. The absorbance was measured at 600 nm by using a microplate reader (BioTek Instrument, USA). The minimum dilution showing no growth indicated the MIC value of strain PD9 CFS against MRSA ATCC 33742.

### Minimum Bactericidal Concentration (MBC)

Five microliters of inoculum in the well with no bacterial growth after 24 h of incubation (in the MIC experiment) was spotted onto mannitol salt agar [7.5% (w/v) NaCl2, 1% (w/v) D-Mannitol, 0.0025% (w/v) phenol red (R&M Chemicals, UK). and 1.5% (w/v) bacteriological agar] (Merck, Germany). The plates were incubated at 37°C for 16 to 24 h. The growth of the MRSA was observed; growth of MRSA was interpreted as bacteriostatic, while no growth meant bactericidal.

### *In vitro* growth inhibition assay

The *in vitro* growth inhibition assay was performed as described by Cao et al. [29] with slight modifications. In each flask, 1 ml of the strain PD9 cell suspension with final cell density 10^3^ cfu/ml, 10^4^ cfu/ml, 10^5^ cfu/ml and 1 ml of MRSA ATCC 33742 suspension with final cell density 10^3^ cfu/ml were independently inoculated into 98 ml of nutrient broth. The mixture was then incubated at 37°C with shaking at 200 rpm. Shake flask with MRSA ATCC 33742 only was used as control. Cell growth of MRSA ATCC 33742 was measured using mannitol salt agar at 24 h interval by using the serial dilution-agar plate method.

### Physicochemical analysis

#### Preparation of cell-free supernatant

Strain PD9 at final cell density of 10^7^ cfu/ml was grown in nutrient broth at 37°C with shaking at 200 rpm for 20 h. CFS obtained by centrifugation of bacterial culture at 8 000 x *g* for 10 min was used in each physiochemical analysis which was performed in triplicates.

#### Thermal stability assay

Thermal stability assay was performed as described by Lee et al. [30] with slight modifications. Strain PD9 CFS was treated independently at 40, 60, 80 and 100°C for 30 min. The inhibition activity of the treated sample (100 μl) to the MRSA ATCC 33742 was tested using agar well diffusion assay and the zone of inhibition was recorded. Untreated sample was used as a reference.

#### pH stability assay

Influence of pH on the activity and stability of strain PD9 CFS was determined as described by Lee et al. [30] with slight modifications. Briefly, the CFS was mixed with 50 mM of sodium acetate (pH 4–6), potassium phosphate (pH 6–8), Tris-CI (pH 8–9), glycine-OH (pH 9–11) and sodium hydrogen phosphate (pH 11–12) at 1:1 ratio. The mixtures were incubated for 2 h at room temperature (24°C). The inhibition activity of the treated sample (100 μl) to the MRSA ATCC 33742 was tested using agar well diffusion assay and the zone of inhibition was recorded. CFS mixed with dH_2_O at ratio 1:1 was used as a reference.

#### Enzyme stability assay

The enzyme stability assay was performed as described by Shekh and Roy [31] with slight modifications. Fifteen microliters of proteinase K (900 U/ml) were added to 1 ml of CFS and incubated for 0.5, 1.0, 1.5 and 2.0 h at 37°C and 55°C respectively. After incubation, each sample was heat inactivated at 70°C for 10 min. Then, 100 μl of treated CFS was tested for antimicrobial activity against MRSA ATCC 33742 by using agar well diffusion assay and the zone of inhibition was recorded. Untreated sample with the addition of 15 μl of dH_2_O was used as a reference.

#### Effects of surfactant and metal ions

The strain PD9 CFS were tested with surfactant: sodium dodecyl sulphate (SDS), Tween 20, Tween 80 and Triton X-100 at final concentration of 1% (v/v) and metal salts: MgCI_2_, NaCI_2_, CuSO_4_, NiSO_4_, KCI, ZnNO_3_ at final concentration 1 mg/ml [32]. Treated samples were incubated for 2 h at room temperature (24°C). Then, 100 μl of the treated CFS were tested for antimicrobial activity against MRSA ATCC 33742 by using agar well diffusion assay and the zone of inhibition was recorded. Untreated sample was used as a reference.

### Extraction and purification of antimicrobial metabolite

Solvent extraction method was performed as described by Bordoloi et al [33] with slight modifications. The strain PD9 culture was mixed with 1-butanol at 5:1 ratio and incubated for 1 h at 37°C with shaking at 200 rpm. The mixture was centrifuged for 10 min at 8000 x *g*. The organic layer formed was collected and evaporated using a rotary evaporator. Antimicrobial activity of the extract obtained was tested against MRSA ATCC 33742 by using disc diffusion assay. The extract obtained was subjected to silica gel column chromatography. The 1-butanol extract was prepared with minimum amount of dichloromethane (DCM). The sample was loaded to a silica gel (G60) column (Merck, Germany) and eluted by eluent with various ratios of DCM and methanol (1:1, 3:1, 5:1, 7:1 and 10:1). About 25–26 fractions were collected. The disc diffusion method was adopted to evaluate the antimicrobial activities of all the fractions eluted against MRSA ATCC 33742. The active fraction was evaporated and diluted in glycine-NaOH buffer for SDS-PAGE analysis.

### Tricine SDS-PAGE and zymogram

Molecular weight of the anti-MRSA metabolite was determined by using tricine SDS-PAGE with stacking gel pH 6.8 (4%), overlaid 1-cm spacer gel pH 8.5 (10%) and separating gel pH 8.5 (16%) as described by Schägger [34] with slight modifications. The electrophoresis was carried out under constant current supply at 100 V for 75 min. After electrophoresis, one part of the gel was fixed with a solution containing 25% ethanol and 5% formaldehyde for 30 min. The gel was washed with distilled water for 3 h and washed again three times with 0.1% tween 80 for 40 min at room temperature. The gel was aseptically overlaid into nutrient agar containing 10^7^ cfu/ml of MRSA ATCC 33742. The plate was incubated at 37°C for 24 h and band with antibacterial activity was observed. The other part of the gel was stained using a staining solution made up of 45% (v/v) methanol, 10% (v/v) acetic acid and 1% (w/v) Coomassie Brilliant Blue R250 for 10–15 min. The protein bands which appeared on the gel was observed.

### HPLC analysis

The active fraction obtained from the silica gel column chromatography was introduced into a reverse phase high performance liquid chromatography (RP-HPLC) system (Perkin Elmer, USA) using C18 reverse phase column (Perkin Elmer, USA). The separation was performed by the gradient method using two solvents: A (95% dH_2_O and 5% acetonitrile (Merck, Germany) and B (100% acetonitrile). The flow rate of the mobile phase was 1 ml min^-1^. The elution gradient was as follows: 0% solvent B for 3 minutes, 0–40% solvent B for 5 min, 40–60% solvent B for 5 min, 60–80% solvent B for 5 min, 80–100% solvent B for 5 min and then back to 100% solution A. The purity of the peptide was determined by the number of peaks which appeared on the chromatogram. The peak was collected and tested against MRSA ATCC 33742.

### Statistical analysis

In order to determine the statistical relationship among the antibacterial activities of the tested bacteria, a one-way analysis of variance (ANOVA) test was used (NCSS: Statistical Software,LLC.,Kaysville, Utah, USA) [35]. The results were considered significant if P values were < 0.05. All reported values were expressed as mean values obtained from three replicates (n = 3), unless stated otherwise.

## Results

### Identification of bacterial strains using *gyrB*

**Fig 1:**
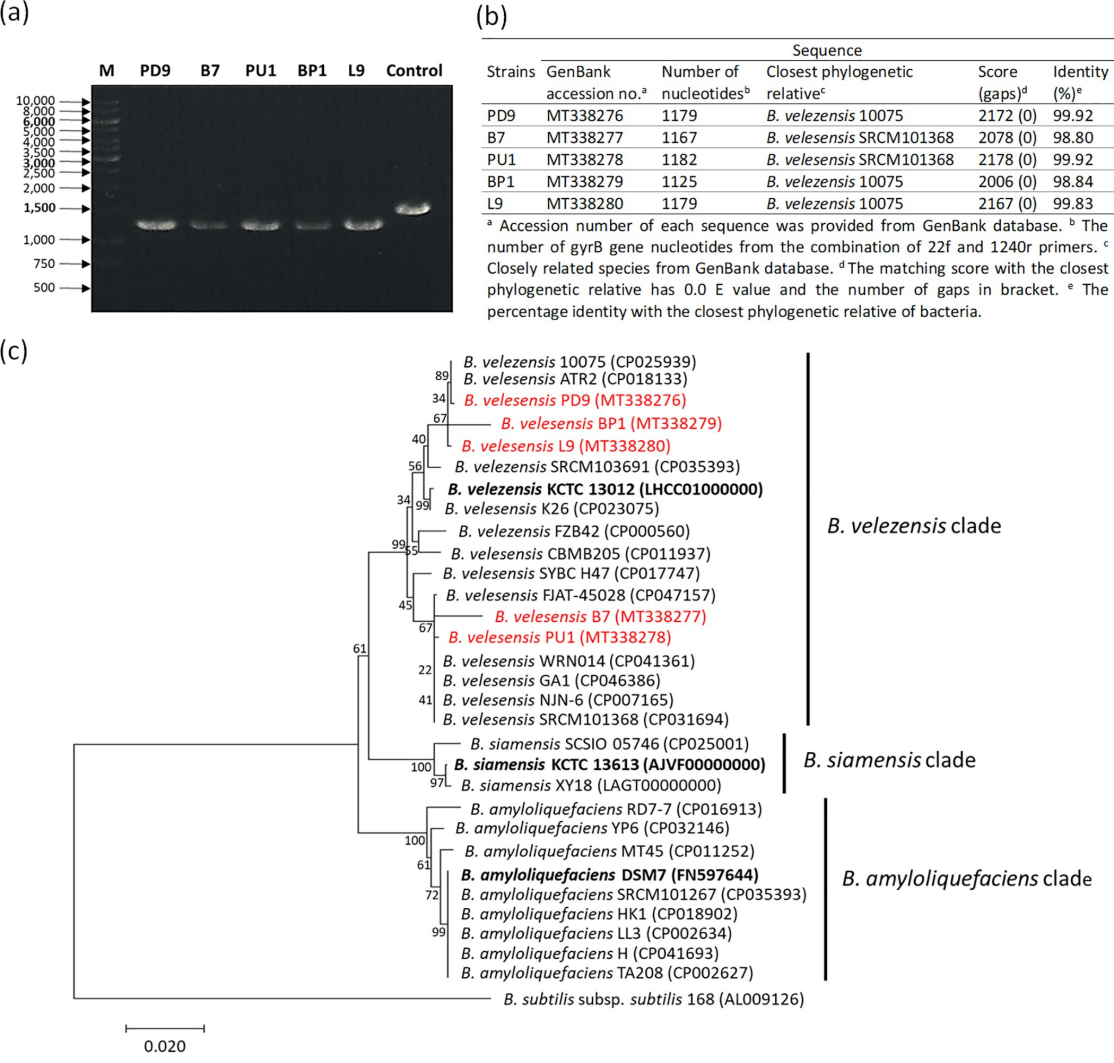
Analysis of *gyrB* sequence. (a) The *gyrB* (approximately 1,200 bp) of *B*. *velezensis* strains were amplified using PCR. The bacterial strains are labelled at the top of the gel photo. “M” = Marker GeneRuler 1 kb Plus DNA ladder (Thermo Fisher Scientific, USA). (b) The comparison of *gyrB* sequences of *B*. *velezensis* strains with the *gyrB* sequence in GenBank. (c) The *gyrB* phylogenetic analysis within the *B*. *amyloliquefaciens* operational group. Type strains are in bold and the bacterial strains used in this study are in red. *B*. *subtilis* subsp. *subtilis* 168 was chosen as an outgroup.

An approximately 1.2-kbp partial fragment of *gyrB* was amplified from the bacterial genomic DNA for reassessment of bacterial strain identity (**Fig 1**). The amplified fragments were sequenced and compared to the sequences available in the GenBank database. The *gyrB* sequence of the bacterial strains showed ≥ 98% similarity to the closest known species in the database. The *gyrB* sequences were used for the phylogenetic analysis. The results demonstrated that strains PD9, B7, PU1, BP1 and L9 belonged to the *B*. *velezensis* clade and thus were closely related to *B*. *velezensis*. All the bacterial strains *gyrB* sequences had been deposited in the NCBI database under the accession numbers: MT338276 (*B*. *velezensis* PD9), MT338277 (*B*. *velezensis* B7), MT338278 (*B*. *velezensis* PU1), MT338279 (*B*. *velezensis* BP1) and MT338280 (*B*. *velezensis* L9). All the bacterial strains were subjected to antimicrobial activity screenings.

### Antagonistic effect of *B*. *velezensis* against MRSA strains and other pathogens

The antagonistic activities of the *B*. *velezensis* strains against MRSA strains were determined (**Table 1**). All strains of *B*. *velezensis* showed inhibitory activities against all the MRSA strains tested including the MSSA strain ATCC 12228. Overall, strains ATCC 33741, ATCC 33742 and ATCC 700699 were highly inhibited by all *B*. *velezensis* strains. The inhibitory activities of all the *B*. *velezensis* strains observed against MSSA strain ATCC 12228 were quite similar to those with the tested MRSA strains. Strains PD9 and BP1 showed high inhibition activities against all tested MRSA strains and MSSA strain ATCC 12228. The biggest zone of inhibition recorded was from strain PD9 against MRSA 33742 (21.0 ± 0.4 mm) and the lowest zones of inhibition recorded were from strains PU1 and B7 against MRSA ATCC 25922 (11.0 ± 0.5 mm). It was noted that there was no significant difference in the inhibitory effects of the *B*. *velezensis* strains towards different MRSA strains. Therefore, a study on the activity of strain PD9 against MRSA ATCC 33742 was chosen for further analyses.

**Table 1:** Antibacterial activities of *B*. *velezensis* strains against several pathogenic bacteria.

Reference bacteria		Inhibition zone exhibited by *B*. *velezensis* strains (mm)				
		PD9	B7	PU1	BP1	L9
*Staphylococcus aureus*						
	MSSA strain ATCC 12228	14.7 ± 0.8	12.3 ± 0.9	11.7 ± 0.4	15.0 ± 0.8	13.0 ± 0.8
	MRSA strain ATCC 25922	14.3 ± 0.4	11.0 ± 0.5	11.0 ± 0.5	14.3 ± 0.4	11.7 ± 0.8
	MRSA strain ATCC 33741	19.5 ± 0.3	18.5 ± 0.5	19.0 ± 0.5	12.4 ± 0.3	16.5 ± 0.3
	MRSA strain ATCC 33742	21.0 ± 0.4	19.0 ± 0.3	20.0 ± 0.5	17.3 ± 0.5	20.0 ± 0.3
	MRSA strain ATCC 700699	14.7 ± 0.4	16.7 ± 0.4	15.3 ± 0.5	18.7 ± 0.4	18.7 ± 0.4
Gram-positive						
	*Clostridium perfringens*	ND	ND	ND	ND	ND
	*Bacillus cereus*	15.0 ± 0.7	10.5 ± 0.7	15.5 ± 0.7	10.0 ± 0.2	17.5 ± 0.7
Gram-negative						
	*Serratia marcescens*	ND	ND	ND	ND	ND
	*Vibrio parahaemolyticus*	24.5 ± 0.7	24.0 ± 0.7	22.0 ± 0.7	20.0 ± 0.5	21.0 ± 0.7
	*Vibrio alginolyticus*	13.0 ± 0.7	13.5 ± 0.5	13.5 ± 0.5	11.0 ± 0.7	12.5 ± 0.7
	*Pseudomonas aeruginosa*	ND	ND	ND	ND	ND
	*Aeromonas hydrophila*	17.0 ± 0.5	17.0 ± 0.7	18.0 ± 0.7	14.5 ± 0.7	15.0 ± 0.5
	*Alcaligenes faecalis*	15.3 ± 0.3	ND	10.0 ± 0.7	ND	ND

The *B*. *velezensis* strains CFS (served as crude antimicrobial compounds) were used for agar well diffusion assay. Reported values are mean ± standard deviation, “ND” = non-detectable and CFS = cell-free supernatant.

The cell-free supernatant of *B*. *velezensis* strains were also used for antimicrobial screenings against several pathogenic bacteria including Gram-positive, and Gram-negative bacteria (**Table 1**). All *B*. *velezensis* strains showed broad-spectrum inhibitions across different groups of pathogenic bacteria. Strains PD9 and PU1 were able to inhibit the growth of *Bacillus cereus*, *Vibrio parahaemolyticus*, *V*. *alginolyticus*, *Aeromonas hydrophila* and *Alcaligenes faecalis*. Strains B7, BP1 and L9 showed similar patterns of inhibition to the strains PD9 and PU1, except against *Alcaligenes faecalis*.

### Minimum Inhibitory Concentration (MIC) and Minimum Bactericidal Concentration (MBC) of strain PD9 cell-free supernatant

**Fig 2:**
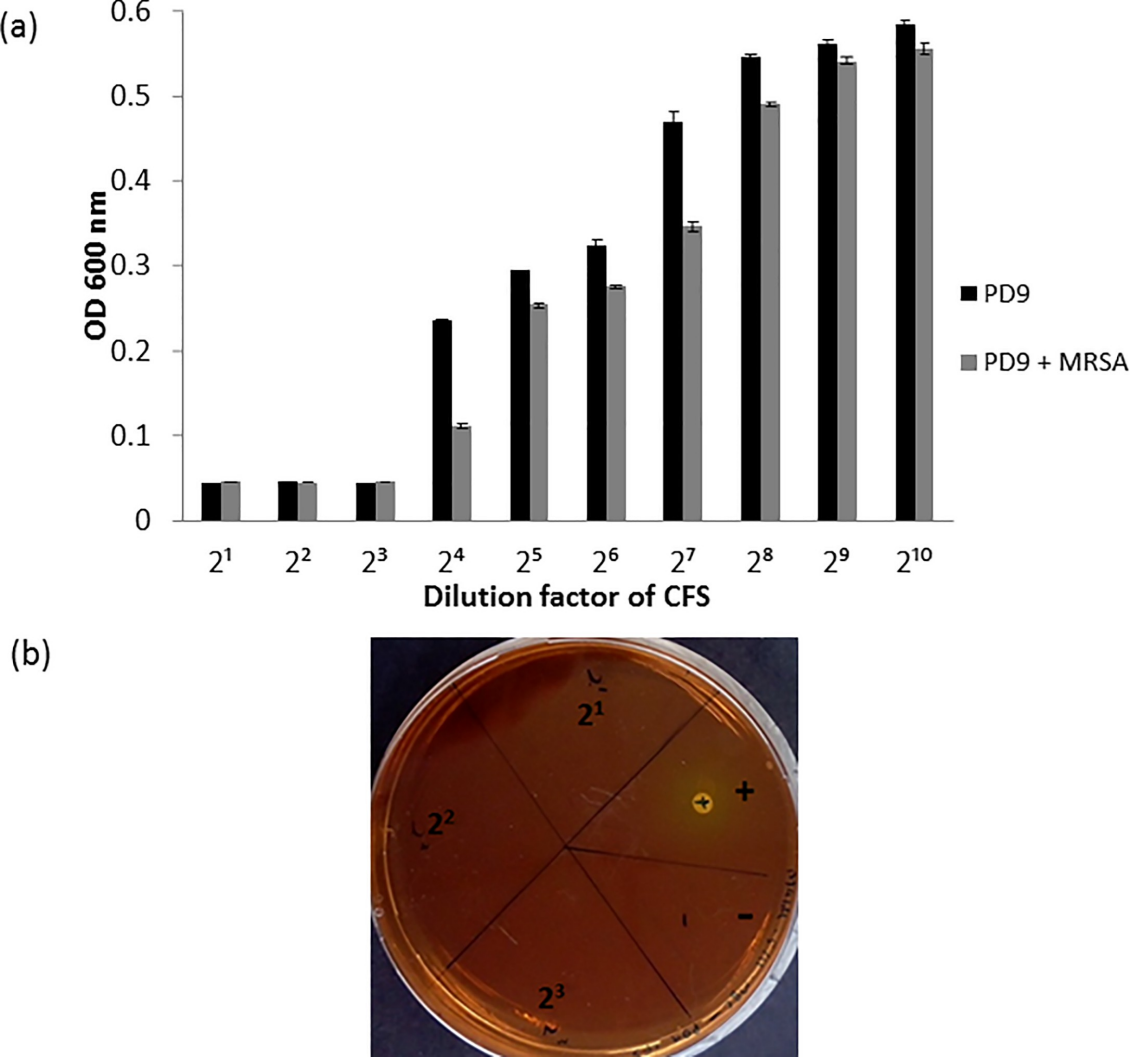
Determination of MIC and MBC values of *B*. *velezensis* strain PD9 CFS on MRSA ATCC 33742. (a) The determination of MIC value. Growth of MRSA ATCC 33742 in nutrient broth inoculated with different dilutions of CFS from (i) strain PD9 and (ii) strain PD9 + MRSA ATCC 33742, after 16 h incubation. Un-inoculated nutrient broth serves as negative control. Reported values are mean ± standard deviation (error bar). (b) The determination of MBC value. Bactericidal activity of strain PD9 CFS from dilution factors of 2^1^ to 2^3^ on MRSA ATCC 33742 observed on mannitol salt agar after 24 h incubation. MIC = minimum inhibitory concentration, MBC = minimum bactericidal concentration and CFS = cell-free supernatant.

The MIC value of CFS from (i) 20 h-grown strain PD9 culture and (ii) 20 h-grown strain PD9 and MRSA ATCC 33472 (co-culture) were determined. In both samples, no growth of MRSA ATCC 33742 was observed in the wells with CFS dilution factor of 2^1^ to 2^3^. Growths of MRSA ATCC 33742 were detected in wells with CFS of 2^4^ to 2^10^ dilution factors (**Fig 2**). Therefore, the MIC values for both treatments were at 2^3^ dilution factor (125 μl/ml). After 24 h of incubation, no MRSA ATCC 33742 growth was observed (**Fig 2**), indicating the bactericidal activity of the antimicrobial compound present in the CFS of strain PD9. The MBC values for both treatments were found at 2^3^ dilution factor (125 μl/ml).

### Growth inhibition effect

**Fig 3:**
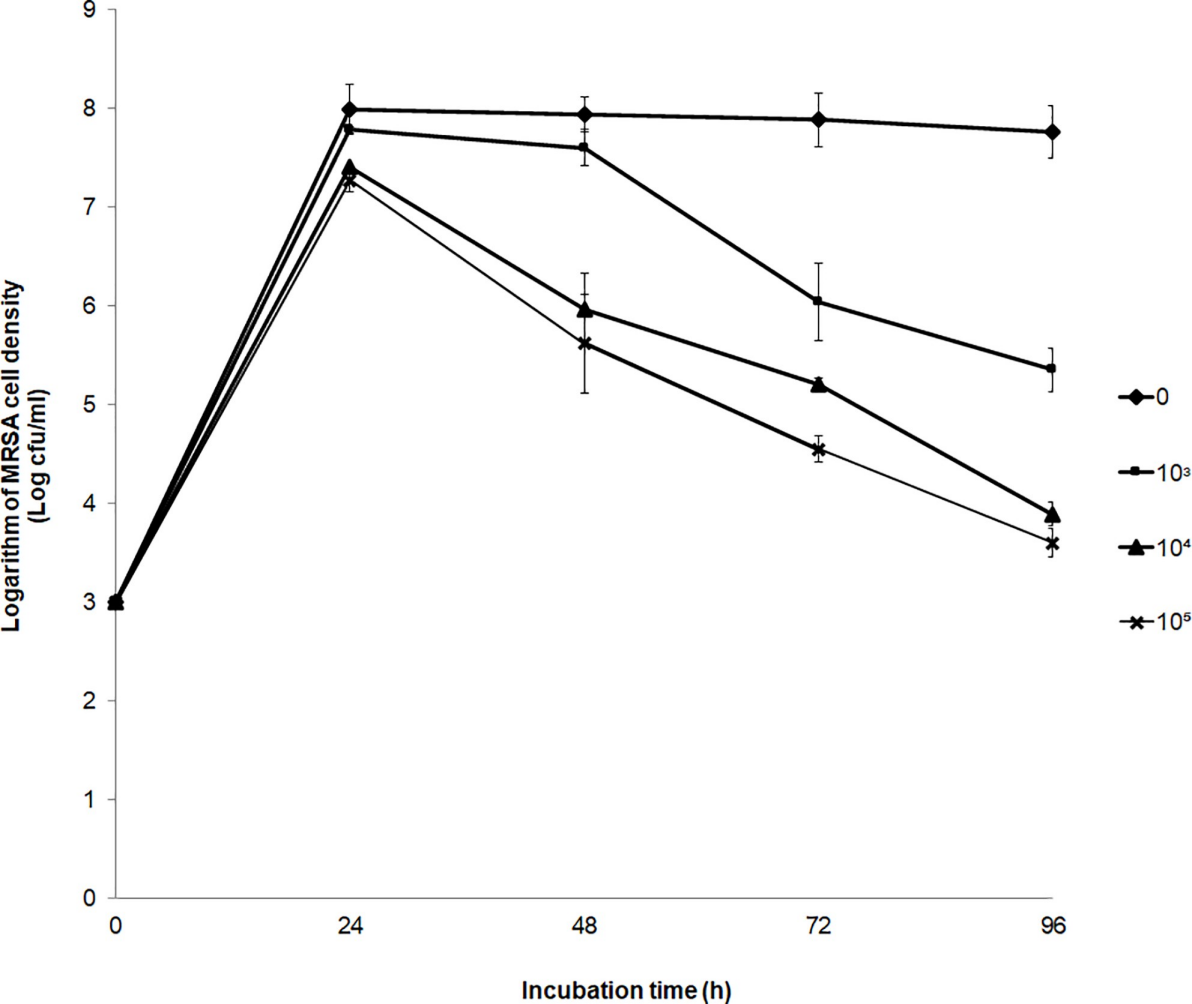
Growth inhibition effect of *B*. *velezensis* strain PD9 on MRSA ATCC 33742. The inhibitory effect of strain PD9 at the final cell density of 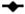: 0 cfu/ml (control), 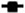: 10^3^ cfu/ml, 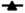: 10^4^ cfu/ml, and 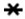:10^5^ cfu/ml on the growth of MRSA ATCC 33742. Reported values are mean ± standard deviation (error bar).

The growth inhibition effect of strain PD9 on MRSA ATCC 33742 was determined. The growth of MRSA ATCC 33742 was reduced as compared to the control when strain PD9 was inoculated at final cell densities of 10^3^ to 10^5^ cfu/ml (**Fig 3**). The results indicated that the higher density of strain PD9 inoculated in the culture, the higher the reduction of MRSA ATCC 33742 growth. Also, the longer the incubation, the lower the growth of MRSA ATCC 33742. The logarithm of MRSA ATCC 33742 cell densities at 10^3^,10^4^ and 10^5^ cfu/ml was reduced to 31.05, 49.87 and 53.60%, respectively as compared to the control (100%) after 96 h of incubation (**Fig 3**). Based on the results, strain PD9 could reduce the growth of MRSA ATCC 33742 in co-culture growth condition.

### Physicochemical analyses

#### Thermal, pH and enzyme stability of the crude antimicrobial compound

**Fig 4:**
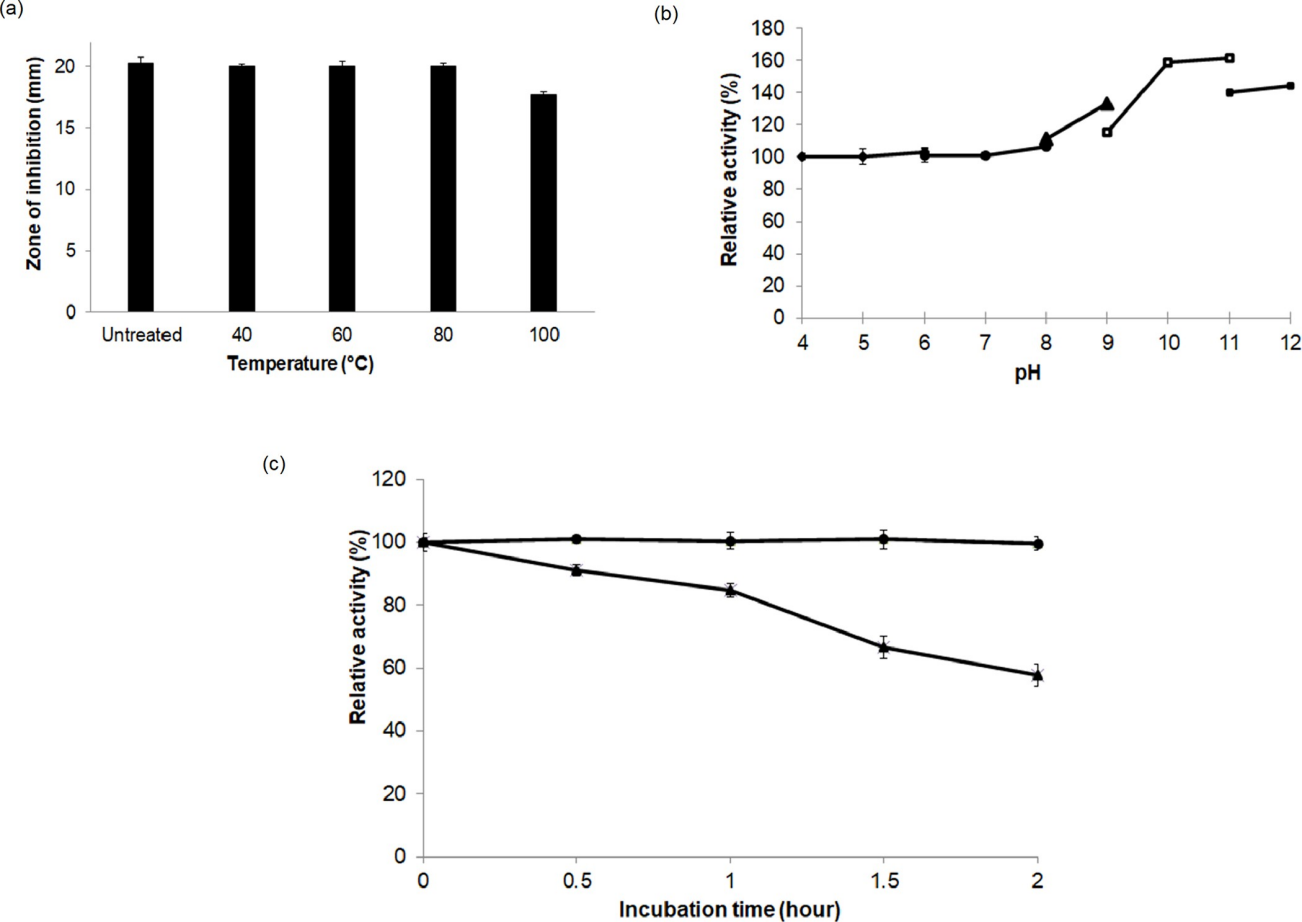
Physicochemical analyses of strain PD9 CFS on MRSA ATCC 33742 lawn cell plate. (a) Thermal stability of strain PD9 CFS on MRSA ATCC 33742 lawn cell plate after the heat treatment. Strain PD9 CFS was treated at 40 to 100°C for 30 min. The inhibitory activity was tested using agar well diffusion assay. CFS at room temperature served as a control. (b) pH stability of strain PD9 CFS on MRSA ATCC 33742 lawn cell plate after 30 min of pH treatment. The buffers used for the treatments were 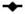 sodium acetate (pH4-6), 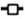 sodium phosphate (pH 6–8), 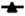 Tris-HCI (pH 8–9), 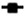 glycine-OH (pH 9–11) and 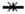 sodium hydrogen phosphate (pH 11–12). The buffer alone served as a negative control. (c) Enzyme stability of strain PD9 CFS on MRSA ATCC 33742 lawn cell plate after strain PD9 CFS was treated with proteinase K for 0.5 to 2.0 h at 37°C (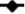) and 55°C (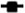). CFS without proteinase K treatment served as a control. Reported values are mean ± standard deviation (error bar); CFS = cell-free supernatant.

The antimicrobial activity of the *B*. *velezensis* strain PD9 CFS was not affected at temperatures of 40 to 80°C. There was no significant difference (P<0.05) in the inhibitory activity of the strain PD9 CFS to MRSA ATCC 33742 when incubated at different temperatures for 30 min (**Fig 4**). However, the inhibitory activity slightly declined at 100°C. There was a 15% reduction as compared to the control (untreated). The result showed that the antimicrobial compound present in the CFS was highly stable in a wide range of temperatures. The retained activity at 80°C indicated that the compound was thermally stable.

The strain PD9 CFS was treated with different pH values of buffer at 1:1 ratio. Based on the clearing zone measured, there was no significant difference in the inhibitory activities at pH 4–7. The relative zones of inhibition values were similar to the untreated sample (**Fig 4**). However, the relative activity was higher at pH 8–12. The highest activity was observed when the CFS was treated at pH 10 and 11. The results showed that the antimicrobial compound present in CFS was stable at various pH values and was not affected at acidic and neutral conditions. Interestingly, inhibitory activity was enhanced at alkaline condition.

Treatment with proteinase K was done to test the stability of the strain PD9 CFS to the enzyme. Based on the zone of inhibition measured, the activity was not affected when the CFS was treated with proteinase K at 37°C. However, the inhibitory activity slightly declined when CFS was treated at 55°C for 0.5 to 2.0 h (**Fig 4**). The inhibitory activity declined up to 8.92, 15.32, 33.33 and 42.34% after 0.5, 1.0, 1.5 and 2.0 h treatments, respectively.

#### Effects of surfactants and metal ions

The strain PD9 CFS was treated independently with different types of surfactants and metal ions. Based on the clearing zones observed, there was no significant difference (P<0.05) in the inhibitory activities of the CFS treated with a majority of the tested surfactants and metal ions (**Table 2**). However, there were slight decline of the inhibitory activities of CFS treated with Triton X-100 and with MgCl_2_. Triton X-100 and MgCl_2_ reduced the inhibitory activities of CFS by 8.35 and 6.48%, respectively.

**Table 2:** Effect of surfactants and metal ions on inhibitory activities of strain PD9 CFS on MRSA ATCC 33742.

Treatment		Inhibition zone (mm)
Surfactant 1% (v/v)		
	Control	20.0 ± 0.5
	Tween 20	19.3 ± 0.3
	Tween 80	19.3 ± 0.3
	Triton X-100	18.3 ± 0.5
	SDS	19.7 ± 0.4
Metal ion (1 mg/ml)		
	Control	20.7 ± 0.3
	MgCI_2_	19.3 ± 0.5
	NaCI_2_	20.0 ± 0.3
	ZnNO_3_	20.3 ± 0.3
	CuSO_4_	19.7 ± 0.5

The CFS was treated with surfactants and metal salts for 2 h at room temperature. CFS without treatment served as a control. Reported values are mean ± standard deviation; CFS = cell-free supernatant.

## Extraction and purification of anti-MRSA peptide from strain PD9

**Fig 5:**
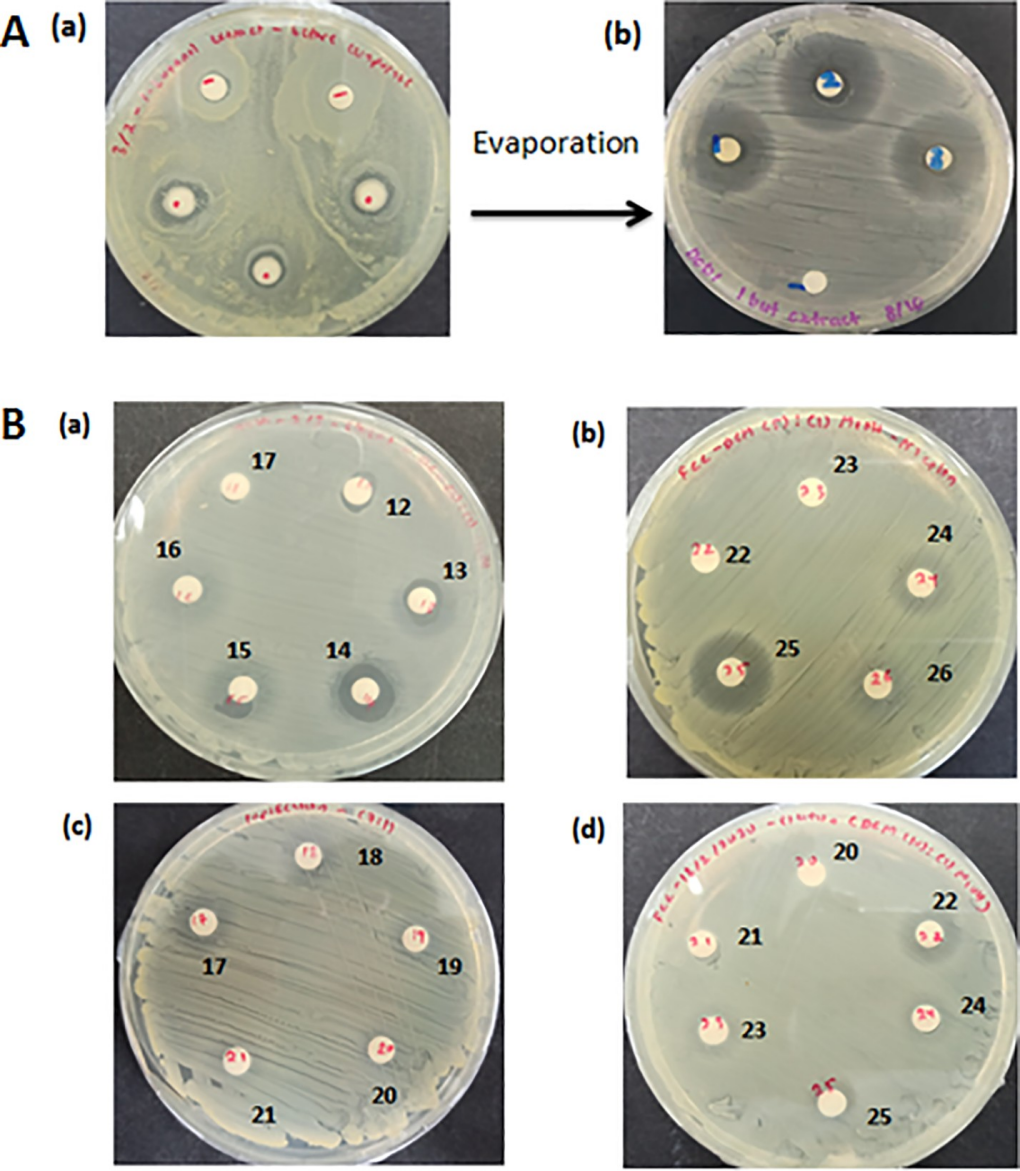
Antimicrobial activity of the 1-butanol extract and the silica gel purified product. (A) Antimicrobial activity of the 1-butanol extract observed (a) before and (b) after evaporation on MRSA ATCC 33742 lawn cell plate. The antimicrobial compound from the 24-h cell culture of strain PD9 was extracted using 1-butanol as the organic solvent. (B) Antimicrobial activity of the eluted fractions from different ratios of DCM: MeOH observed on MRSA ATCC 33742 lawn cell plate. (a) Active fractions from eluent with ratio DCM (3):(1) MeOH. (b) Active fractions from eluent with ratio DCM (5):(1) MeOH. (c) Active fractions from eluent with ratio DCM (7):(1) MeOH. (d) Active fractions from eluent with ratio DCM (10):(1) MeOH.

**Fig 6:**
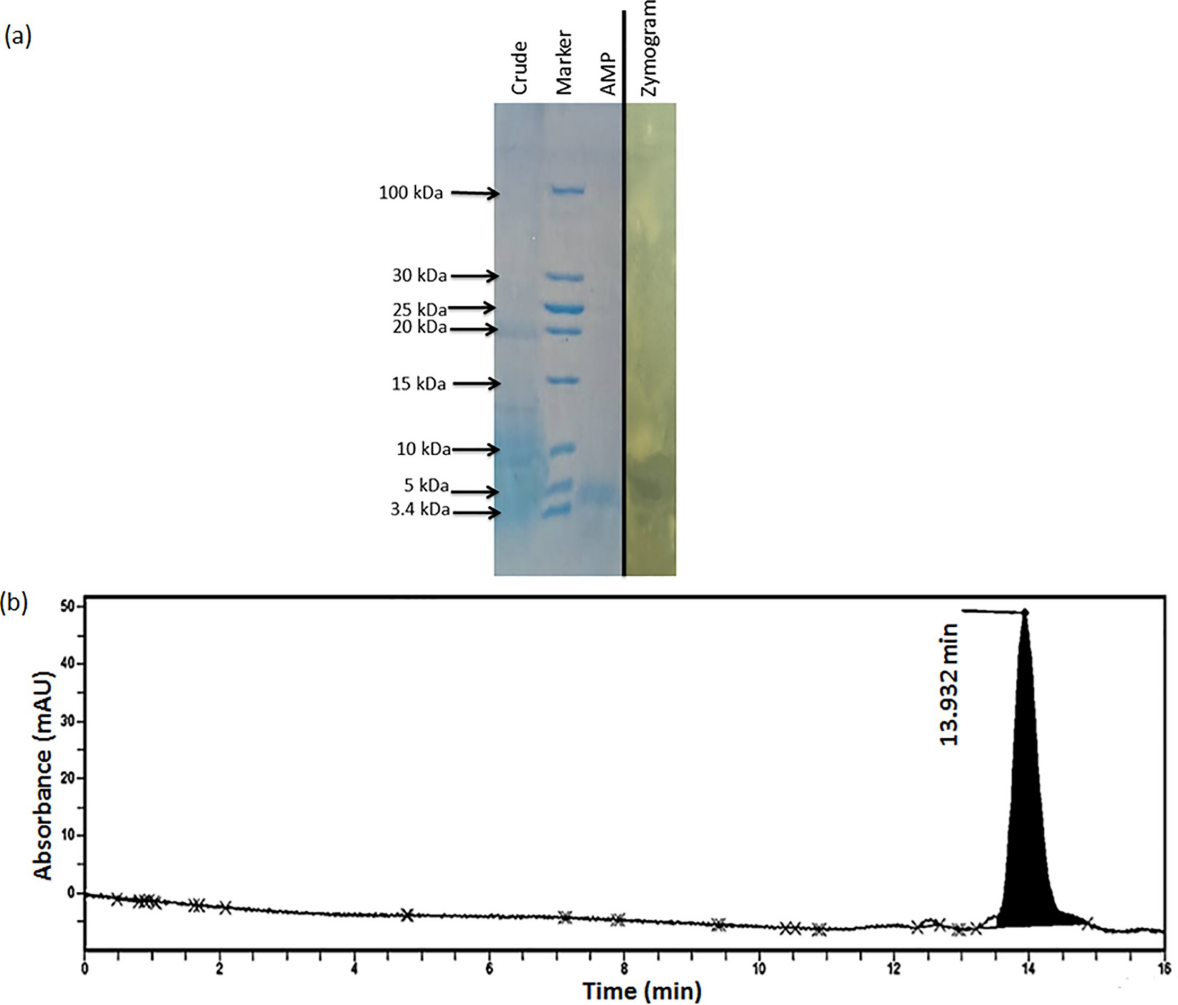
Analysis of silica gel purified *Bacillus velezesis* PD9 antimicrobial peptide using tricine SDS-PAGE and HPLC. A) Tricine–SDS-PAGE analysis and direct detection of antimicrobial activity of the antimicrobial peptide using zymogram. The single band of purified peptide observed on SDS-PAGE with a halo zone observed against MRSA ATCC 33742 on the zymogram after 24 h of incubation. Vertical black lines in between the AMP and zymogram represents different gels (S1 and S2 Figs) merged together to show the direct inhibitory activity of AMP against MRSA ATCC 33742. Marker: Unstained low range protein ladder. AMP: Antimicrobial peptide. (B) HPLC analysis of the antimicrobial peptide. A single peak was observed on the chromatogram indicating the purity of the silica gel purified antimicrobial peptide indicating successful purification of the antimicrobial peptide.

The extracellular metabolite of strain PD9 was extracted using 1-butanol as the organic solvent. The antimicrobial activities of the 1-butanol extract before and after evaporation were tested against MRSA ATCC 33742. The zones of inhibition observed before and after evaporation were 12.7±0.8 mm and 26.3±0.3 mm, respectively (**Fig 5**). The 1-butanol extract obtained were subjected to silica gel column chromatography. The antimicrobial activities of the eluted fractions from different ratios of DCM:MeOH (1:1, 3:1, 5:1, 7:1 and 10:1) were tested against MRSA ATCC 33742. The fractions eluted from the eluents with ratios of DCM:MeOH (3:1, 5:1, 7:1 and 10:1) exerted antimicrobial activities (**Fig 5**). Only the eluent with ratio of DCM:MeOH (1:1) did not show any inhibition activity. The eluent with ratio 5:1 showed the highest inhibition activity and three active fractions were collected. Among the three fractions, fraction number 24 showed the biggest zone of inhibition (19.8±0.5 mm). The active fractions obtained from the silica gel purified product were used for HPLC analysis. A single peak was observed on the chromatogram at retention time 13.932 min (**Fig 6**). The results indicated that the purified product only contained a single anti-MRSA compound. The eluted peak showed inhibitory activity against MRSA ATCC 33742 (result not shown).

### Molecular weight of the anti-MRSA peptide

The 1-butanol extract and silica gel purified products were subjected for SDS-PAGE analysis to determine the molecular size of the antimicrobial peptide. As depicted in **Fig 6** the crude extract showed multiple protein bands, whereas the purified product showed a single band on SDS-PAGE. The purified product produced a halo zone against MRSA ATCC 33742 on the zymogram. The band with antibacterial activity against MRSA ATCC 33742 showed a molecular mass of approximately \~5 kDa.

## Discussion

Exploration of antimicrobial metabolites from natural sources such as bacteria is considered as one of the most promising alternatives to combat the infection of pathogens in humans, animals and plants [9, 11, 36–38]. Based on the previous studies, the *Bacillus subtilis* complex group which is comprised of *B*. *subtilis*, *B*. *amyloliquefaciens*, *B*. *licheniformis*, *B*. *velezensis* and *B*. *pumilus*, is one of the most common groups of bacteria associated with antimicrobial metabolite production [10, 39]. Initially, five bacterial strains (PD9, B7, PU1, BP1 and L9) which belonged to the *B*. *amyloliquefaciens* operational group were isolated from stingless bee products and identified on the basis of 16SrRNA [18]. However, the classification of the bacterial species within *B*. *subtilis* complex group based on 16S rRNA is considered as being not enough due to the highly conserved nature of the gene [10, 19, 20, 40]. The bacterial species in this group are not able to be classified using 16S rRNA gene analysis alone. Thus, their species level identifications based on 16S rRNA analysis are often inconsistently assigned in NCBI database [19, 41]. Therefore, confirmation of the identity of these five strains were done by using another phylogenetic marker namely *gyrB*. Based on the blast of *gyrB* sequences in the GenBank database and the phylogenetic tree analysis, all the strains were identified as *B*. *velezensis*. These results were expected as *B*. *velezensis* was previously classified under the sub-group of “*B*. *amyloliquefaciens* operational group” under the *B*. *subtilis* complex group [19]. *B*. *velezensis was* also often recognized as being synonymous to *B*. *amyloliquefaciens* based on phylogenomic analysis [42].

All the strains of *B*. *velezensis* were able to inhibit MRSA strains (MSSA ATCC 12228, MRSA ATCC 700699, MRSA ATCC 25922, MRSA ATCC 33741 and MRSA ATCC 33742). However, the inhibitory effects were slightly different across the strains. The results indicated that the antimicrobial compound produced could be used as a universal therapeutic agent against different strains of MRSA. This inhibitory activity against MRSA was further explored as MRSA strains are considered as one of the most common antibiotic resistant pathogens in the world [43]. In addition, *B*. *velezensis* strains were subjected to the antimicrobial activity assay against various Gram-positive and Gram-negative bacteria. All strains of *B*. *velezensis* especially strains PD9, B7 and PU1 showed a broad-spectrum of inhibition across the different types of bacteria tested. This could be due to the production of diverse types of antimicrobial compounds. As had been reported, *B*. *velezensis* encoded high genetic capacity for antimicrobial compound production such as of polyketides (bacillaene, difficidin and macrolactin), cyclic lipopeptides (bacillibactin, surfactin, bacillomycin-D and fengycin) and others [44].

For further evaluation, the inhibitory activity of strain PD9 against MRSA ATCC 33742 was used as a reference. MIC was determined by using the CFS of strain PD9 culture and CFS was obtained from mixed strain PD9 and MRSA ATCC 33742 cultures. The MIC and MBC values obtained for both treatments were at 125 μl/ml (**Fig 2**). In another related study, bacterial supernatant from *Lactobacillus* strains isolated from commercial yogurt showed MIC values of 50 to 128 μl/ml against several pathogens including *S*. *aureus*, *S*. *typhii* and *E*. *coli* [45]. Similar values were obtained for MIC and MBC in another study of *L*. *casei* against *P*. *aeruginosa*, where both values reported were 62.5 μl/ml (2^4^ dilution factor) [46]. However, there is no report found on the MIC and MBC values of bacterial supernatant from *B*. *velezensis* against MRSA. Based on the result obtained, the growth of MRSA ATCC 33742 was lower (higher antimicrobial activity) when MRSA ATCC 33742 was treated with CFS from strain PD9 + MRSA ATCC 33742 mix cultures. These results indicated that, in the presence of the pathogen (MRSA ATCC 33742), strain PD9 produced more antimicrobial metabolites. The induction of antimicrobial metabolite in the presence of competing species or pathogens was also observed in related studies of *B*. *subtilis* B38 against MRSA [47] and soil bacterial isolates against *E*. *coli* and *S*. *aureus* [48]. From the MBC analysis, it was found that the CFS had bactericidal activity. The bactericidal activities of the anti-MRSA compound were also reported in other related studies. For example, cyclic dipeptide: cyclo(L-leucyl-L-prolyl) from *B*. *amyloliquefaciens* MMS-50, antimicrobial peptide from *B*. *subtilis* URID 12.1 and anti-MRSA compound from marine *Streptomyces* exhibited bactericidal activities against MRSA [14, 49, 50].

The *in vitro* effect of strain PD9 on the growth inhibition of MRSA ATCC 33742 was determined. A significant reduction of MRSA ATCC 33742 was observed after 48 h of incubation after the inoculation of strain PD9 at high final cell density (10^4^ and 10^5^ cfu/ml). This could be due to the higher amounts of antimicrobial metabolite produced by the higher cell density [29].

For further understanding of the characteristics and more information on the expected antimicrobial metabolite present in strain PD9 CFS, physicochemical analyses were performed. The effects of temperature, pH, proteinase K, metal ions and surfactant treatments were tested. The antimicrobial compounds were stable at high temperatures as well as at acidic and alkaline pH. The property of thermal stability would be useful during industrial process handling. Heat stable property was also reported in cyntibiotics (class II bacteriocins). These are the small peptides (<10 kDa) containing one or more disulfides bond that are necessary for their activities [8]. In this study, the inhibitory activity of strain PD9 CFS was improved at alkaline condition. Also, it is high likely that the antimicrobial compound from strain PD9 is neither an organic acid nor a phenolic compound. This is due to the instability of organic acids and phenolic compounds at neutral and alkaline conditions [32, 45], In addition, the inhibitory activity of strain PD9 CFS was reduced after proteinase K treatment at 55°C indicating the proteinaceous nature of the antimicrobial metabolite. The stability of the strain PD9 antimicrobial metabolite at a wide range of temperatures and pH values as well as the decrease in activity after proteinase K treatment resembled the features of several antimicrobial metabolites such as bacteriocin from *B*. *velezensis* BS2, amysin from *B*. *amyloliquefaciens* SP-1-13LM and an antimicrobial peptide from *B*. *firmus* H_2_O-1 [16, 51, 52]. The inhibitory activity of the antimicrobial metabolite was retained when treated with metal ions and surfactants. These results showed that metal ions were neither inhibitors nor co-factors to the antimicrobial metabolite. Both metal ions and surfactants did not cause any adverse effect nor improve the biological activity of CFS. Similar results were also observed for the antimicrobial peptide produced from *B*. *subtilis* strains URID 12.1 and RLID 12.1 [32, 49] as well as for the bacteriocins produced from *Streptococcus thermophilus* [53]. Based on the physicochemical analysis, the antimicrobial compound present in the strain PD9 CFS might be a peptide antibiotic, such as bacteriocins, lipopeptide, glycopeptides or cyclic peptides and its stability in a wide range of temperatures and pH values is well suited for industrial purposes.

In this study, the anti-MRSA metabolite was successfully extracted using 1-butanol as the organic solvent. Higher inhibitory activity (bigger zone of inhibition) of the extract was observed after the evaporation process. The antimicrobial metabolite was purified using silica gel column chromatography. To further evaluate the purity of the silica gel purified product, HPLC analysis was performed. A single peak appeared on the chromatogram which indicated the success of using silica gel column chromatography for the purification process of the anti-MRSA peptide. The method successfully eliminated other unwanted metabolites or proteins in the extracts. The size of the purified anti-MRSA peptide obtained was approximately ~5 kDa. Although, there are reports on antimicrobial peptides with size of ~5 kDa isolated from *B*. *subtilis* 168 [54], *B*. *cereus* BC7 [55] and *Candida intermedia* LAMAP1790 [56], to the best of our knowledge, this is the first report of an extracellularly produced antimicrobial peptide with the similar size from *B*. *velezensis* which exhibits anti-MRSA activity. Another anti-MRSA metabolite produced by *B*. *velezensis* was identified as an aminoglycoside with a molecular weight around 418.32 Da [11].

## Conclusion

*B*. *velezensis* strains from stingless bee products are important bacteria that can produce antimicrobial metabolites with inhibitory activities against MRSA strains and various types of pathogenic bacteria. *B*. *velezensis* strain PD9 produces an anti-MRSA metabolite with high thermal and pH stabilities. The present characterizations reveal the interesting properties of the antimicrobial peptide which show its promising potential for controlling pathogenic MRSA strains. Further efforts on identification, structural elucidation and characterization as well as mechanism studies are needed to allow for clinical applications of the anti-MRSA peptide from this bacterial strain.

## Supporting information

S1 Fig Raw image of Tricine SDS-PAGE for Fig 6. AMP is the purified antimicrobial peptide from *Bacillus velezensis* PD9.
(DOCX)

S2 Fig Raw image of zymogram for Fig 6. AMP is the purified antimicrobial peptide from *Bacillus velezensis* PD9. Halo zone was observed on the antimicrobial peptide band indicating MRSA growth inhibition.
(DOCX)
