# Supplementary material for: Antimicrobial activities of Bacillus velezensis strains isolated from stingless bee products against methicillin-resistant Staphylococcus aureus
Source: PLoS One. 2021 May 11;16(5):e0251514. doi: 10.1371/journal.pone.0251514 (PMC8112681; doi:10.1371/journal.pone.0251514)
Supplement: S1 Fig — AMP is the purified antimicrobial peptide from Bacillus velezensis PD9. (DOCX) [file pone.0251514.s001.docx]

Marker

Crude

AMP

X X


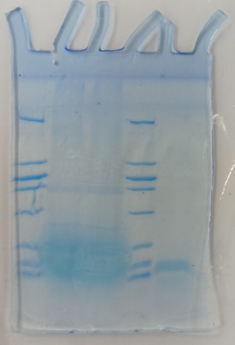


10 kDa

30 kDa

5 kDa

3.4 kDa

15 kDa

20 kDa

25 kDa

100 kDa

**S1 Fig. Raw image of Tricine SDS-PAGE for Fig 6**. AMP is the purified antimicrobial peptide from *Bacillus velezensis* PD9.
