# Supplementary material for: Antimicrobial activities of Bacillus velezensis strains isolated from stingless bee products against methicillin-resistant Staphylococcus aureus
Source: PLoS One. 2021 May 11;16(5):e0251514. doi: 10.1371/journal.pone.0251514 (PMC8112681; doi:10.1371/journal.pone.0251514)
Supplement: S2 Fig — AMP is the purified antimicrobial peptide from Bacillus velezensis PD9. Halo zone was observed on the antimicrobial peptide band indicating MRSA growth inhibition. (DOCX) [file pone.0251514.s002.docx]

X X AMP


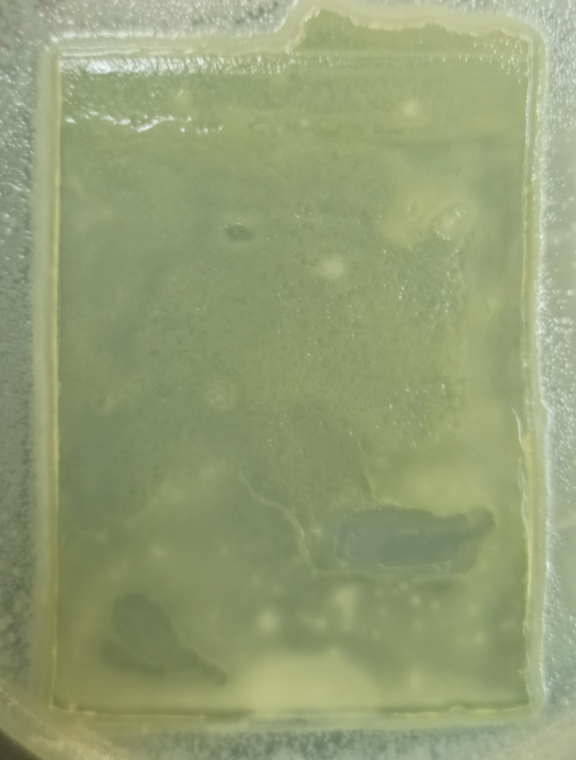


Anti-MRSA peptide

**S2 Fig. Raw image of zymogram for Fig 6**. AMP is the purified antimicrobial peptide from *Bacillus velezensis* PD9. Halo zone was observed on the antimicrobial peptide band indicating MRSA growth inhibition.
